# Poorer sleep quality in patients with chronic temporomandibular disorders compared to healthy controls

**DOI:** 10.1186/s12891-022-05195-y

**Published:** 2022-03-14

**Authors:** Yeon-Hee Lee, Q-Schick Auh, Jung-Sub An, Tae Kim

**Affiliations:** 1 Department of Orofacial Pain and Oral Medicine, Kyung Hee University Dental Hospital, #26 Kyunghee-daero, Dongdaemun-gu, Seoul, 02447 South Korea; 2 Department of Orthodontics, Seoul National University Dental Hospital, Seoul, South Korea; 3 Department of Biomedical Science and Engineering, Gwangju Institute of Science and Technology, Gwangju, South Korea

**Keywords:** Sleep quality, Chronic, Temporomandibular disorder, Pittsburgh sleep quality index, STOP-Bang, Epworth sleepiness scale

## Abstract

**Objectives:**

This study aimed to investigate and compare sleep quality between patients with chronic temporomandibular disorder and healthy controls, and to analyze the association of sleep quality with disease characteristics, obstructive sleep apnea risk factors, and excessive daytime sleepiness.

**Methods:**

Chronic temporomandibular disorder patients (*n* = 503, mean age: 33.10 ± 13.26 years, 333 females) and 180 age- and sex-matched healthy controls (mean age: 32.77 ± 12.95 years, 116 females) were included, who completed well-organized clinical report and answered questions on sleep quality (Pittsburgh Sleep Quality Index), sleep apnea risk factors (STOP-Bang questionnaire), and excessive daytime sleepiness (Epworth sleepiness scale).

**Results:**

Mean global Pittsburgh Sleep Quality Index scores were significantly higher in the patients (6.25 ± 2.77) than in healthy controls (3.84 ± 2.29) (*p* <  0.001). Poor sleep was significantly more prevalent in the patient group (56.9%) than in healthy controls (22.2%) (*p* <  0.001). Compared with healthy controls, chronic temporomandibular disorder patients had a higher likelihood of obstructive sleep apnea (STOP-Bang total score ≥ 3; 7.2% vs. 16.1%; *p* <  0.01) and higher excessive daytime sleepiness (Epworth sleepiness scale score ≥ 10; 12.8% vs. 19.7%; *p* <  0.05). Age (odds ratio = 2.551; *p* <  0.001), female sex (odds ratio = 1.885; *p* = 0.007), total Epworth sleepiness scale score (odds ratio = 1.839; *p* = 0.014), and headache attributed to temporomandibular disorder (odds ratio = 1.519; *p* = 0.049) were the most powerful predictors of poor sleep (global Pittsburgh Sleep Quality Index score ≥ 5) in chronic temporomandibular disorder patients.

**Conclusion:**

Chronic temporomandibular disorder patients had markedly impaired sleep quality than healthy controls. Poorer sleep in patients with chronic temporomandibular disorder was associated with a variety of clinical factors, including a higher likelihood of excessive daytime sleepiness, older age, female gender, higher Epworth sleepiness scale scores, and the presence of headache attributed to temporomandibular disorder.

## Introduction

Humans require good sleep, in both quality and quantity, and sufficient pain control mechanisms. Sleep is essential for physical health, emotional well-being, brain functioning, daytime performance, and pain control [1]. Abnormal pain modulation system can cause chronic pain, and chronic pain is associated with sleep problems [2, 3]. Patients with chronic pain have poorer sleep than healthy controls in terms of sleep latency, sleep efficiency, and awakenings after sleep onset [4]. Thus, reciprocal, bidirectional interactions exist between chronic pain and sleep disorders, deterioration in either of them can ultimately become comorbid conditions [5]. In clinical settings, sleep problems have been found to impact 88% of patients with chronic pain [6]. Contrarily, more than 40% of patients who have sleep-related problems report chronic pain [7]. The prevalence of chronic pain ranges from 10 to 40% [8], similar to that of sleep disorders, ranging from 10 to 36% [9].

Temporomandibular disorders (TMDs) are quite common chronic orofacial pain conditions. TMDs are highly prevalent, affecting up to 25% of the population, with a peak incidence at 20–40 years of age, and 1.5–2 times more prevalent in female than in male [10]. Pain is the most common symptom of TMDs [11], which can affect areas such as the ears, eyes and/or throat, frequently causing neck pain and headache, and involve disturbances in mandibular movement, and functional impairment. Sleep problems are common in patients with TMD, with approximately 90% reporting poor sleep quality [12]. Deterioration of sleep quality and impairment of sleep structure occur in a significant proportion in TMD patients and are thought to be a risk factor for maintaining and worsening symptoms [13–15], but their impact is not clearly known in chronic TMD. Sleep quality in patients with TMD decreases as the number of diagnoses of painful TMD increases based on the research diagnostic criteria for TMD (RDC / TMD) Axis I [13]. Although several studies have been conducted on sleep quality in TMD patients [12, 16, 17], few researchers use the updated diagnostic criteria for TMD (DC / TMD).

Chronic TMD can present persistent, recurrent, or chronic pain associated with TMJ and/or muscles involved in the masticatory system [11], which leads to highly disabling. As the etiology of chronic TMD is considered multifactorial, chronic TMD has an idiopathic basis in which the pathophysiology mechanism is not well understood. Patients with chronic TMD tend to undergo a process of central sensitization, central hyperexcitation, resulting in plastic changes in neurons at the spinal and/or supraspinal level [18, 19]. These changes could lead to an alteration in the descending pathways of pain modulation [20]. Through these processes, pain inhibition can be deficient in chronic TMD patients, and they can have pain even with non-noxious stimuli, or suffer from hyperalgesia and rest pain. In addition, chronic TMD is also accompanied by pain amplification due to neuroendocrine dysfunction, and sleep problems and psychological disorders are common comorbidities [21, 22]. TMD patient ratings of poor sleep are associated with increased clinical pain severity and psychological distress [12]. Sleep bruxism is considered an aggravating factor for TMD pain, but the relationship remains controversial [23, 24]. However, there is little evidence to indicate that chronic TMD patients have poor sleep quality, and it is not known which factors clearly affect the sleep quality of chronic TMD patients.

In the present study, three sleep questionnaires including the Pittsburgh Sleep Quality Index (PSQI), Epworth sleepiness scale (ESS), and the snoring, tiredness, observed apnea, high blood pressure (STOP)-BMI, age, neck circumference, and male gender (Bang) questionnaire were used to investigate factors influencing sleep quality and quantity in chronic TMD patients. The Pittsburgh Sleep Quality Index (PSQI) is a valid, reliable, and internationally known instrument for assessing self-perceived sleep quality [25]. Excessive daytime sleepiness (EDS) is a common symptom in sleep disorders, headaches, and chronic pain [26, 27]. The EDS status can be evaluated with the ESS, and 28.6% of TMD patients presented with EDS based on this questionnaire [27]. Obstructive sleep apnea (OSA) is a sleep breathing disorder characterized by repeated agitation of nocturnal breathing disruption caused by upper airway collapse, and is considered as a putative risk factor for TMD [28]. For screening for OSA, STOP-Bang questionnaire with high reliability has been used in the clinical field [28, 29]. The first attempt was made in which three questionnaires were simultaneously applied to chronic TMD diagnosed by DC/TMD.

This cohort study aimed to evaluate clinical characteristics and sleep-related factors in patients with chronic TMD, to determine which parameters were significantly associated with decreased sleep quality in them. Our findings in this study will support the usefulness of an integrated model of demographics and disease characteristics in explaining sleep quality deterioration in chronic TMD patients.

## Materials and methods

### Participants

To investigate the research purpose, the authors designed and implemented a retrospective cohort study conducted at the Department of Orofacial Pain and Oral Medicine at Kyung Hee University Dental Hospital of Seoul. The research protocol was reviewed in compliance with the Helsinki Declaration and approved by the Institutional Review Board of the Kyung Hee University Dental Hospital (KHD IRB no. 1804–2). Written informed consent was obtained from all individual participants.

Of the patients who had visited the Department of Orofacial Pain and Oral Medicine between June 1, 2018, and November 30, 2019, the study sample was composed of chronic TMD patients according to inclusion and exclusion criteria. Inclusion criteria of chronic TMD patients were as follows: completed a set of routine TMJ assessments, as well as constructive questionnaires, no treatment of the current episode other than medication, and a history of current TMD symptoms lasting more than 6 months. Patients who were pregnant or had a history of systemic osteoarthritis, rheumatoid arthritis, other connective tissue diseases, general infection, neurological/neuropathic diseases, and those under 18 years of age were excluded from the study. Healthy controls were recruited through advertisements in the hospital. Inclusion criteria of healthy controls were as follows: systemic good health, no history of macrotrauma or surgery on TMJ and neck area, no previous diagnosis of TMD, no TMD signs and symptoms, and an absence of TMD on brief clinical examination. Healthy individuals who satisfies the following inclusion criteria and exclusion, and who are sex- and age-matched with chronic TMD patients were finally selected. All individuals included in the study completed three questionnaires including PSQI, ESS, and STOP-Bang.

### TMD classification and clinical evaluation

Clinical evaluation procedures included an oral examination, interview, panoramic radiography, and a comprehensive questionnaire in DC/TMD Axis I diagnostic algorithms for TMD diagnoses [30]. A subtype of diagnostics included myofascial pain, disc displacement, arthralgia, and headache attributed to TMD based on DC/TMD Axis I diagnostic algorithms.

We diagnosed patients who experienced TMJ pain for more than six months after onset as chronic TMD. When the pain persisted more than three to six months, individuals were usually considered in a chronic state [31]. The intensity of TMD pain was measured using a visual analog scale (VAS) (0–10, 10 being the worst possible pain). The duration of symptoms in the masticatory muscles, TMJ, and adjacent structures was reported by patients in how many days elapsed since the patient evert felt TMD-related symptoms. Information concerning patient demographics included age, sex, height, body weight for body mass index (BMI = weight/height^2^), and neck circumference were collected by a research assistant. We described sleep quality differences between chronic TMD patients and healthy controls, investigated subgroup of TMD influences, and studied sleep quality in the full set of DC/TMD subgroups of Axis I.

### Sleep quality evaluation using PSQI

PSQI, a sleep self-rated questionnaire, was employed to measure sleep quality. Habitual sleep quality and sleep disturbance in the past month were assessed using the 19-item PSQI, a well-validated self-report questionnaire. The PSQI has seven components that concern subjective sleep quality, sleep latency, sleep duration, sleep efficiency, sleep disturbances, use of sleep medication, and daytime dysfunction. Each subscale is weighted equally, scored from 0 (good sleep/no problems) to 3 (poor sleep/severe problems), summing to a global PSQI score (range, 0–21). Higher scores denote worse sleep quality, and a global score > 5 has a diagnostic value in distinguishing poor from good sleep [32].

### The risk evaluation of obstructive sleep apnea (OSA) with STOP-Bang

To complementarily evaluate the factors related to OSA and EDS along with sleep quality obtained from PSQI, STOP-Bang and ESS questionnaire were additionally used. The STOP-Bang questionnaire is validated screening tool for identifying high likelihood for OSA. The STOP-Bang questionnaire includes eight dichotomous (yes/no) questions related to these clinical features of sleep apnea. For each question, answering “yes” scores 1, a “no” response scores 0, and the total score ranges from 0 to 8. The exposure of interest was a binary-low or high likelihood for OSA; the low likelihood of OSA: Yes to < 3 questions, high likelihood of OSA: Yes to ≥3 questions [33]. All patients were asked to complete the STOP-Bang questionnaire.

### Excessive daytime sleepiness measured by ESS

The ESS is a validated clinical tool for the evaluation of excessive daytime sleepiness (EDS) [34]. Unlike other scales, which measure sleepiness at a single time point, the ESS is designed to evaluate the general level of sleepiness. The ESS is an eight-item, self-administered questionnaire designed to provide a measure of the subject’s propensity to fall asleep in a variety of situations. The subject is instructed to answer how likely it is that he/she would fall asleep in those different situations, by giving a score on a 4-point scale (0–3). Thus, the total score (the sum of scores of the eight items) of the ESS ranges from 0 to 24. The higher the score, the greater the possibility the individual will fall asleep during the daytime. The ESS total scores were dichotomized into scores ≤10 and > 10; the latter is considered to be clinically significant EDS [35]. We used a score of 10 or higher on the ESS to measure excessive sleepiness.

### Statistical analysis

The data were analyzed using SPSS Statistics for Windows, Version 20.0 (IBM Corp., Armonk, NY, USA). Continuous variables are presented as means and standard deviations (SD), and categorical variables are presented as frequencies and percentages. Differences between groups were examined by using the chi-square test for categorical variables and t-test and one-way analysis of variance (ANOVA) with Tukey post-hoc test for numeric variables. Three questionnaires included in this study were for sleep quality, risk for OSA, and daytime sleepiness. To understand the risk for poor sleep quality, we performed a multiple logistic regression analysis to determine the relative risk for poor sleep (PSQI global score of ≥5). In addition, dichotomous variables based on cut-off points of each questionnaire’s global scores were used as dependent or independent variables of logistic regression analysis. The high likelihood of OSA (STOP-Bang total score ≥ 3), and EDS (ESS score of > 10) (independent variables), as well as age, sex, and symptom duration, were taken into consideration simultaneously to predict a value of a dependent variable (poor sleep) for chronic TMD patients. For all analyses, a two-tailed level of statistical significance of a *p*-value was set at less than 0.05.

## Results

### General description

In this period, 525 patients who were diagnosed with chronic TMD were included, and 22 patients were excluded because they lacked medical documentations. Finally, 503 patients (mean age: 33.10 ± 13.26 years, 333 females) were designated as the chronic TMD group. One hundred and eighty age- (mean age: 32.77 ± 12.95 years, 116 females) and sex-matched TMD-free volunteers were designated to the healthy control group.

Table 1 presents the demographic characteristics of chronic TMD patients compared to healthy controls. Chronic TMD was more prevalent in females (66.2%) than in males (43.8%), and the female:male was 1.51:1. BMI scores were significantly higher in chronic TMD than in healthy controls (22.32 ± 3.57 vs. 21.45 ± 2.76, *p* = 0.001), and the mean values were in the normal range. In chronic TMD patients, the mean VAS score of chronic TMD patients was 4.89 ± 2.45, and the mean symptom duration was 589.74 ± 1315.01 days.

**Table 1:** Demographic characteristics of chronic TMD patients compared to healthy controls

	Chronic TMD patients (***n*** = 503)	Healthy control (***n*** = 180)	p-value
**Parameter**	**n (%) or Mean ± SD**	**n (%) or Mean ± SD**	
**Age (years)**^**a**^	33.10 ± 13.26	32.77 ± 12.95	0.771
**Age groups**			< 0.001*** 10s < 20s < 30s < over 40
Under 20 years old (10s) ^**b**^	61 (17.59 ± 2.28)	20 (17.60 ± 1.56)	
20–30 years old (20s)	208 (24.58 ± 2.86)	77 (24.43 ± 2.61)	
31–40 years old (30s)	93 (34.46 ± 3.09)	35 (34.03 ± 3.00)	
More than 40 years old (over 40)	141 (51.49 ± 7.41)	48 (51.55 ± 6.87)	
**Sex (Female %)**^**c**^	333 (66.2)	116 (64.4)	0.367
**BMI (kg/m**^**2**^**)**^**a**^	22.32 ± 3.57	21.45 ± 2.76	0.001**
**VAS**	4.89 ± 2.45	–	**n.a.**
**Symptom duration (days)**	589.74 ± 1315.01	–	**n.a.**

*TMD *temporomandibular disorder, *BMI *body mass index, *SD *standard deviation, *n.a. *not available

A *p*-value < 0.05 was considered significant.^**^: *p*-value <0.01. ^***^: *p*-value <0.001

^a^: The results were obtained via t- test, ^b^ : The mean difference among age subgroups was obtained by one-way analysis of variance and post-hoc analysis. ^c^: The results were obtained from χ2 test

### Differences in PSQI global scores and poor sleep between TMD group and controls

Table 2 presents the results of the subjective sleep quality in terms of PSQI score and their comparison of PSQI between chronic TMD patients and healthy controls. PSQI components, including subjective sleep quality, sleep latency, sleep duration, sleep efficiency, sleep disturbances, and use of sleep medication, were significantly impaired in patients than in healthy controls (all *p* <  0.05). Interestingly, the PSQI global scores were significantly higher in chronic TMD patients than in healthy controls (6.25 ± 2.77 vs. 3.84 ± 2.29, *p* <  0.001). In other words, subjective sleep quality of the chronic TMD group was more impaired than the healthy control group. The proportion of poor sleepers was significantly higher in chronic TMD patients than in healthy controls (56.9% vs. 22.2%, *p* <  0.001). A total of 56.9% of chronic TMD patients were poor sleepers, and the prevalence was significantly higher in chronic TMD patients than in controls (22.2%) (*p* <  0.001).

**Table 2:** Comparison of Pittsburgh Sleep Quality Index (PSQI) between chronic TMD patients and healthy controls

	Chronic TMD patients (***n =*** 503)	Healthy control (***n =*** 180)	*p*-value
**Parameter**	**n (%) or Mean ± SD**	**n (%) or Mean ± SD**	
**PSQI**			
**Component 1: Subjective sleep quality (0–3)** ^**a**^	1.50 ± 0.79	0.59 ± 0.61	< 0.001***
**Component 2: Sleep latency (0–3)** ^**a**^	0.83 ± 0.97	0.42 ± 0.72	< 0.001***
**Component 3: Sleep duration (0–3)** ^**a**^	0.72 ± 0.91	0.61 ± 0.79	0.035*
**Component 4: Sleep efficiency (0–3)** ^**a**^	0.38 ± 0.79	0.11 ± 0.36	< 0.001***
**Component 5: Sleep disturbances (0–3)** ^**a**^	1.20 ± 0.63	0.45 ± 0.60	< 0.001***
**Component 6: Use of sleep medication (0–3)** ^**a**^	0.15 ± 0.54	0.04 ± 0.28	0.001**
**Component 7: Daytime dysfunction (0–3)** ^**a**^	1.41 ± 0.90	1.79 ± 1.04	< 0.001***
**PSQI global score (0–21)** ^**a**^	6.25 ± 2.77	3.84 ± 2.29	< 0.001***
**Poor sleeper (PSQI global score ≥ 5)** ^**b**^	286 (56.9)	40 (22.2)	< 0.001***

*TMD* temporomandibular disorder, *SD *standard deviation

A *p*-value < 0.05 was considered significant. *: *p*-value < 0.05, **: *p*-value < 0.01, ***: *p*-value < 0.001

^a^: The results were obtained via t- test. ^b^: The results were obtained from χ2 test

### Disease characteristics and their relationship with PSQI in chronic TMD patients

Table 3 summarizes the clinical factors and subgroups of TMD diagnoses associated with impaired sleep quality. For the chronic TMD patients, both the PSQI global score (6.56 ± 2.79 vs. 5.65 ± 2.64, *p* < 0.001) and the poor sleeper ratio (62.8% vs. 45.3%, *p* < 0.001) was significantly higher in females than in males. Conversely, in healthy control, both the PSQI global score (3.85 ± 2.12 vs. 3.83 ± 2.59, *p* < 0.944) and the poor sleeper ratio (25.0% vs. 20.7%, *p* = 0.506) were not significantly higher in females than in males.

**Table 3:** Descriptive statistics of disease characteristics and their relationship with Pittsburgh Sleep Quality Index (PSQI) in chronic TMD patients

		PSQI		Poor sleepers (PSQI > 5)	
Parameter	Subgroup n (%)	PSQI global score	*p*-value^**a**^	Poor sleeper n (%)	*p*-value^**b**^
**Demographics of TMD patients (*n*** **= 503)**					
**Age groups**					
Under 20 years old (10s)	61 (12.1)	4.69 ± 2.34	< 0.001***	20/61 (32.8)	< 0.001***
20–30 years old (20s)	208 (41.4)	5.85 ± 2.45	10s < 20s = 30s = over 40^**c**^	104/208 (50.0)	
31–40 years old (30s)	93 (18.5)	6.87 ± 2.91	20s < 30s = over 40^**d**^	61/93 (65.6)	
More than 40 years old (over 40)	141 (28.0)	7.11 ± 2.90		101/141 (71.6)	
**Sex**					
Male	170 (33.8)	5.65 ± 2.64	< 0.001***	77/170 (45.3)	< 0.001***
Female	333 (66.2)	6.56 ± 2.79		209/333 (62.8)	
**TMD Axis I diagnosis in TMD patients***(Multiple diagnoses are allowed per patient)*					
**Myofascial pain**					
**[presence]**	**465 (92.4)**	**6.31 ± 2.82**	0.023*	268/465 (57.6)	0.145
**[absence]**	**38 (7.6)**	**5.50 ± 1.98**		18/38 (47.4)	
Disc displacement					
[presence]	278 (55.3)	6.27 ± 2.77	0.862	159/278 (57.2)	0.928
[absence]	225 (44.7)	6.23 ± 2.78		127/225 (56.4)	
Arthralgia					
[presence]	355 (70.6)	6.22 ± 2.88	0.888	203/355 (57.2)	0.448
[absence]	148 (29.4)	6.26 ± 2.72		83/148 (56.1)	
**Headache attributed to TMD**					
**[presence]**	**285 (56.7)**	**6.58 ± 2.85**	0.002**	**178/285 (62.5)**	0.005**
**[absence]**	**218 (43.3)**	**5.82 ± 2.60**		**108/218 (49.5)**	

*TMD* temporomandibular disorder, *PSQI *Pittsburgh Sleep Quality Index, *SD *standard deviation

*p*-Value significance was set at < 0.05. *: *p*-value < 0.05, **: *p*-value < 0.01, ***: *p*-value < 0.001

^a^: The *p*-value for the test of the mean difference of the PSQI global score between subgroups. ^b^: The *p*-value for the comparison results of the number (%) between subgroups via χ2 test and Boneferroni correction. The mean difference among age subgroups was obtained by one-way analysis of variance and post-hoc analysis. ^c^: The PSQI global scores of subgroups over 20 years of age were significantly higher than that of 10s, and the scores of 20s, 30s, and over 40 were not significantly different. ^d^: The PSQI global scores over 30 years of age were significantly higher than that of 20s, and the scores of 30s and over 40 were not significantly different

As for age, an increase in age was associated with an increase in PSQI global scores and the occurrence of poor sleep. When we divided TMD patients into subgroups based on their age, the PSQI global scores were significantly higher and the ratio of patients with poor sleep quality was higher in patients in their 30s, 40s, or older as compared with those in their 20s and younger. The PSQI global score of chronic TMD patients younger than 20 years old was 4.69 ± 2.34, and the poor sleeper ratio was 32.8%, whereas the score in patients in their 40s and older was the highest (7.11 ± 2.90), and 71.6% of them were poor sleepers. In contrast, the PSQI global scores did not differ significant difference by age subgroup in healthy control (10s (*n* = 20): 3.70 ± 2.16, 20s (*n* = 77): 3.90 ± 2.53, 30s (*n* = 35): 3.94 ± 2.41, and over 40 (*n* = 48): 3.75 ± 1.87; *p* = 0.967). The proportion of poor sleeper also showed no significant difference by age subgroup in healthy control (10s: 20.0%, 20s: 23.5%, 30s: 22.9%, and over 40: 20.8%; *p* = 0.905).

In addition, multiple TMD sub-diagnoses were allowed in one patient, and the PSQI global score was higher when patients were diagnosed with myofascial pain (6.31 ± 2.82 vs. 5.50 ± 1.98, *p* = 0.023) or headache attributed to TMD (6.58 ± 2.85 vs. 5.82 ± 2.60, *p* = 0.002) than in patients without the diagnoses. The majority of chronic TMD patients simultaneously suffer from pain of myofascial origin (*n* = 465, 92.4%), arthralgia (*n* = 355, 70.6%), headache attributed to TMD (*n* = 285, 56.7%), and disc displacement (*n* = 278, 55.3%). Therefore, we concluded that it is not appropriate to compare the PSQI global and component scores and presence of poor sleep by subgroup.

### Differences in STOP-Bang and ESS between TMD group and controls

STOP-Bang total scores were significantly higher in TMD patients than in healthy controls (1.77 ± 1.86 vs. 1.53 ± 0.77, *p* = 0.017) (Table 4). By detail, the proportion of snoring (15.7% vs. 4.4%) and high blood pressure (6.4% vs. 1.1%) were significantly higher in chronic TMD patients than in controls (all *p* < 0.05). Of the 503 chronic TMD patients, 420 (83.5%) had felt tired, 170 (33.8%) were men, 80 (15.9%) were aged over 50, 28 (5.6%) had observed sleep apnea, 4 (0.8%) had BMI more than 35 kg/m2, and 29 (5.8%) had larger neck circumference than the reference (male > 17 in., female > 16 in.). The presence of these factors did not differ statistically from the control group. Eighty-one out of 503 chronic TMD patients (16.1%) had a high likelihood of OSA.

**Table 4:** Comparison of STOP-BANG and ESS components between groups.

	Chronic TMD patients (***n =*** 503)	Healthy control (***n =*** 180)	*p*-value
**Parameter**	**n (%) or Mean ± SD**	**n (%) or Mean ± SD**	
**STOP-Bang**			
**Snoring (none = 0, yes = 1)**^a^	**79 (15.7)**	**8 (4.4)**	< 0.001***
Tired (none =0, yes =1) ^a^	420 (83.5)	147 (81.7)	0.324
Observed apnea (none =0, yes =1) ^a^	28 (5.6)	6 (3.3)	0.163
**High blood pressure (none = 0, yes = 1)**^a^	**32 (6.4)**	**2 (1.1)**	0.002**
BMI more than 35 kg/m^2^ (none =0, yes =1) ^a^	4 (0.8)	1 (0.6)	0.604
Age over 50 (none =0, yes =1) ^a^	80 (15.9)	30 (16.7)	0.814
Neck circumference (male > 17 in., female > 16 in.) (none =0, yes =1) ^a^	29 (5.8)	7 (3.9)	0.223
Gender, male (Female =0, male =1) ^a^	170 (33.8)	64 (35.6)	0.367
**STOP Bang total score (0–8)**	**1.77 ± 1.86**	**1.53 ± 0.77**	0.017*
**STOP-Bang total score ≥ 3 (high risk of OSA)**	**81 (16.1)**	**13 (7.2)**	0.001**
**Epworth sleepiness scale**			
Sitting and reading (0–3) ^**b**^	0.94 ± 0.73	0.91 ± 0.64	0.588
Watching TV (0–3) ^**b**^	0.63 ± 0.67	0.61 ± 0.60	0.751
**Sitting, inactive in public place (0–3)**^**b**^	**0.59 ± 0.66**	**0.76 ± 0.73**	0.007**
**As a passenger in a car for an hour without a break (0–3)**^**b**^	**1.19 ± 0.89**	**1.04 ± 0.81**	0.047*
**Lying down to rest in the afternoon when circumstances permit (0–3)**^**b**^	**1.45 ± 0.89**	**1.17 ± 0.84**	< 0.001***
Sitting and talking to someone (0–3) ^**b**^	0.28 ± 0.54	0.33 ± 0.56	0.272
Sitting quietly after a lunch without alcohol (0–3) ^**b**^	1.23 ± 0.85	1.34 ± 0.83	0.107
**In a car, while stopped for a few minutes for traffic (0–3)**^**b**^	**0.42 ± 0.63**	**0.54 ± 0.66**	0.043*
ESS total score (0–24) ^**b**^	6.73 ± 3.62	6.71 ± 3.02	0.952
**ESS total score ≥ 10 (EDS)**^a^	**99 (19.7)**	**23 (12.8)**	0.023*

Values are presented as number (%) and mean ± standard deviation

*TMD *temporomandibular disorder, *SD *standard deviation, *STOP-Bang *The snoring, tiredness, observed apnea, high blood pressure (STOP)-BMI, age, neck circumference, and gender (Bang), *ESS *Epworth sleepiness scale, *EDS *excessive daytime sleepiness

*p-*Value significance was set at <0.05. *: *p*-value < 0.05, **: *p*-value <0.01, ***: *p*-value <0.001

^a^: Comparison results of the number (%) between groups performed with χ2 test and Boneferroni correction. ^b^: The results were obtained via t- test

Ninety-nine out of 503 chronic TMD patients (19.7%) had EDS. Total ESS scores were not significantly different; however, the EDS rate was significantly higher in the chronic TMD group than in controls (19.7% vs. 12.8%, *p* = 0.023).

### PSQI, STOP-Bang, and ESS scores according to the number of TMD/AXIS I diagnosis

**Fig. 1:**
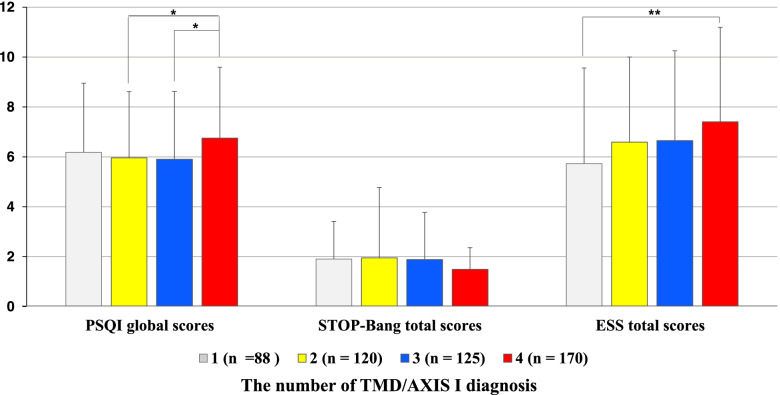
PSQI, STOP-Bang, and ESS scores according to number of TMD/AXIS I diagnosis. Subtypes of TMD diagnosis included myofascial pain, disc displacement, arthralgia using, and headache attributed to TMD. When multiple diagnoses were allowed for each patient, the number of diagnoses the patient had was expressed numerically. 1: In the case of having only one diagnosis among the four diagnoses of TMD based on TMD Axis I (myofascial pain, disc dis-placement, arthralgia, and headache attributed to TMD), 2: having two of the four diagnoses, 3: having three of the four diagnoses, and 4: having all of the four diagnoses. The results were obtained from ANOVA and post-hoc analysis. *: *p*-value <0.05, **: *p*-value <0.01

When multiple diagnoses according to RDC / TMD and DC / TMD was allowed in one patient, 82.5% of chronic TMD patients had two or more multiple TMD subgroup diagnoses, and only 17.5% of the patients had one TMD diagnosis. Even, there were 33.8% (*n* = 170) of patients with four multiple diagnoses. As a result of the ANOVA test, the PSQI global score was significantly higher when chronic TMD patients had four multiple diagnoses (6.75 ± 2.84) than when they had two multiple diagnoses (5.96 ± 2.65) and three multiple diagnoses (5.90 ± 2.72) (*p* = 0.028) (Fig. 1). STOP-Bang total score was not significantly different according to the number of sub-diagnoses. However, in ESS, the ESS total score was significantly higher when chronic TMD patients had four multiple diagnoses (7.40 ± 3.79) than when they had only one sub-diagnosis (5.73 ± 3.83) (*p* < 0.001) (Table 5).

**Table 5:** PSQI, STOP-Bang, and ESS scores according to number of TMD/AXIS I diagnosis

	Number of TMD Axis I diagnosis					
Parameter	1 (***n*** = 88)	2 (***n*** = 120)	3 (***n*** = 125)	4 (*n* = 170)	*p*-value	Post-hoc analysis
**PSQI global scores**	**6.18 ± 2.77**	**5.96 ± 2.65**	**5.90 ± 2.72**	**6.75 ± 2.84**	0.028*	**2 < 4, 3 < 4**
STOP-Bang total scores	1.90 ± 1.51	1.95 ± 2.82	1.88 ± 1.89	1.49 ± 0.87	0.125	
**ESS total scores**	**5.73 ± 3.83**	**6.59 ± 3.41**	**6.65 ± 3.60**	**7.40 ± 3.79**	0.005**	**1 < 4**

Values are presented as mean ± standard deviation. The results were obtained from ANOVA and post-hoc analysis. *p*-Value significance was set at < 0.05. *: *p*-value < 0.05, **: *p*-value < 0.01. TMD: temporomandibular disorder, PSQI: Pittsburgh Sleep Quality Index, STOP-Bang: The snoring, tiredness, observed apnea, high blood pressure (STOP)-BMI, age, neck circumference, and gender (Bang), ESS: Epworth sleepiness scale. When multiple diagnoses were allowed for each patient, the number of diagnoses the patient had was expressed numerically. 1: In the case of having only one diagnosis among the four diagnoses of TMD based on TMD Axis I (myofascial pain, disc dis-placement, arthralgia, and headache attributed to TMD), 2: having two of the four diagnoses, 3: having three of the four diagnoses, and 4: having all of the four diagnoses

### Multivariate logistic regression analysis of factors influencing poor sleep quality

Table 6 presents the significant predictors for poor sleep (PSQI > 5) among chronic TMD patients. To comprehensively examine the risk factors for a poor sleep, multivariate backward stepwise logistic regression analysis of all parameters was performed as the final analysis. The odds ratios (ORs) of each independent variable was interpreted as the change in the incidence of poor sleep in chronic TMD patients. The increase of age (OR = 2.551, 95% CI = 1.662–3.917) was the most powerful predictor for poor sleep, and female sex (OR = 1.885, 95% CI = 1.193–2.978), ESS total score (OR = 1.839, 95% CI = 1.130–2.993), and sub-diagnosis of headache attributed to TMD (OR = 1.519, 95% CI = 1.014–2.308) were putative risk factors for poor sleep. Whereas BMI, VAS, symptom duration, other sub-diagnoses, except for headache, attributed to TMD, STOP-BANG total score did not reach statistical significance.

**Table 6:** Multivariate logistic regression analysis of factors influencing poor sleep (PSQI > 5) among the TMD patients

	PSQI (≤5) (***n*** = 217)	PSQI (> 5) (***n*** = 286)	*p*-value	OR	95% Confidential interval	
Parameter	*n* (%)	*n* (%)			Lower	Upper
**Age [ref. = under average value]**	**56 (25.8)**	**141 (49.3)**	<0.001***	**2.551**	**1.662**	**3.917**
**Female [ref. = male]**	**124 (57.1)**	**209 (73.1)**	0.007**	**1.885**	**1.193**	**2.978**
BMI [ref. = under average value]	90 (41.5)	139 (48.6)	0.146	1.353	0.900	2.036
VAS [ref. = under average value]	121 (55.8)	168 (58.7)	0.951	1.012	0.685	1.495
Duration [ref. = under average value]	61 (28.1)	73 (25.5)	0.711	0.922	0.600	1.418
Myofascial pain by RDC/TMD [ref. = none]	197 (90.8)	268 (93.7)	0.762	1.125	0.523	2.423
Disc displacement by RDC/TMD [ref. = none]	119 (54.8)	159 (55.6)	0.453	0.829	0.508	1.353
Arthralgia by RDC/TMD [ref. = none]	152 (70.0)	203 (71.0)	0.930	0.976	0.576	1.655
**Headache attributed to TMD by DC/TMD**	**107 (49.3)**	**178 (62.2)**	0.049*	**1.519**	**1.000**	**2.308**
STOP-Bang [ref. < 3]	31 (14.3)	50 (17.5)	0.868	1.051	0.584	1.891
**ESS [ref. < 10]**	**34 (15.7)**	**65 (22.7)**	0.014*	**1.839**	**1.130**	**2.993**

*OR* Odds ratio, *CI* Confidential interval, *ESS* Epworth sleepiness scale. Multivariate logistic regression analysis was performed to comprehensively examine the factors affecting poor sleep in chronic TMD patients (*R* = 0.833, R square = 0.787). For obtaining significant results, two-tailed level of statistical significance of a *p*-value was set at less than 0.05

## Discussion

This study comprehensively investigated the clinical characteristics and sleep quality of chronic TMD patients who were diagnosed based on DC/TMD Axis I. The main findings in the present study imply that chronic state TMD patients had poorer sleep than healthy controls, and the magnitude of impaired sleep was associated with increased age, female sex, certain subtypes of TMD diagnosis, including myofascial pain and headache attributed to TMD, and the number of TMD diagnoses in a person. Regarding summary scores and cut-off values of each questionnaire, the presence of EDS was a significant predictor for the poor sleep quality in chronic TMD patients.

The cause of chronic TMD is varied, and its localization, and clinical characteristics are vaguer than with acute pain. Chronic TMD is typically associated with joint dysfunctions such as disc displacement with or without reduction [36], and psychological distress [21]. It is difficult to infer a causal relation between sleep and chronic pain; patients with chronic pain commonly suffer from poor sleep quality [37]. Approximately 45.5% of patients with chronic pain suffer from sleep disorders, and older age was significantly associated with pain experience [9]. In this study, 56.9% of the chronic TMD group met the proposed cut-off of 5 of PSQI for poor sleep, compared to 22.2% of the control group that had impaired sleep. The proportion of TMD patients with poor sleep (56.9%) in the present study is higher than the rate reported among adults with TMD (43.3%) in other studies [15, 38] but lower than those in other studies involving older TMD patients (69.6–90.0%) [12, 39].

The etiology of chronic TMD fundamentally related to peripheral and central factors together. Peripheral factors of TMD include inflammatory processes, including synovitis and myositis, infection, or irritation. Peripheral factors are the main cause of acute pain, but as the pain becomes chronic, the importance of the central factor increases [40]. Central factors include sleep deterioration, impairment of psychological health, and dysfunction of central pain inhibitory system [15, 41]. Furthermore, central sensitization is a key characteristic of chronic pain presented as hypersensitivity, particularly tactile allodynia, hyperalgesia, and enhanced temporal summation [42, 43], which commonly presents in chronic TMD patients. According to a recent meta-analysis focusing on endogenous pain control of orofacial pain including TMD, abnormal endogenous pain control function could be a potential mechanism of pain chronicization [44]. As in other idiopathic pain disorders such as fibromyalgia and irritable bowel syndrome, TMD patients frequently present with overlapping signs and symptoms of sleep disorders [45].

Sleep quality of chronic TMD patients was more impaired by increased age. In the present study, the increase of age (OR = 2.551, 95% CI = 1.662–3.917) was the most powerful predictor for poor sleep, and female sex (OR = 1.885, 95% CI = 1.193–2.978) was followed. In young adults, consolidated sleep at night and wakefulness during the day emerges from a balance between the brainstem, hypothalamus, and midbrain [46]. In older adults, this operation is not effective, and decreased sleep duration, increased sleep latency, impaired sleep quality, shallow sleep, and changes in sleep structure can lead to sustained or deepening pain [47]. In addition, sleep patterns and structures are known to change across the lifespan, with up to 50% of older adults report difficulties initiating and/or maintaining sleep [48]. Chronic sleep disturbances are considered as indications of poor health, vice versa, older adults commonly suffer from pain syndromes, arthritis, hormonal changes, neurodegeneration, psychological distress, cancer, renal and urologic diseases, and medical comorbidities all of which can contribute to sleep disorders [49]. Thus, older adults with chronic TMD are less likely to get enough rest and recovery through sleep than younger ones.

Female sex was also a major contributor to poor sleep quality in chronic TMD patients. There is limited recent evidence of interactions among sex, TMD chronicity, and sleep. However, it has been found that females show higher clinical and experimental pain sensitivity, and worse sleep impairments than males [5]. Few probable causes for poor sleep quality in the female sex may be explained based on sex differences concerning mechanisms of pain of the craniofacial system [50]. Furthermore, contribution of female sex may reflect changes of systems beyond the physical axis of the orofacial area and in line with the biopsychosocial model, blending centrally mediated factors. Especially in postmenopausal female, an increase in sleep problems may be associated with the presence of noticeable hormonal changes, age-associated changes in sleep and psychosocial distress [5]. In clinical research, females reported TMD symptoms, headache, and had muscle tenderness and joint sounds more often than males [51]. It will be crucial to determine whether the effect of sleep on chronic TMD pain, and vice versa, is moderated by key demographic variables, such as age or sex.

Considering the EDS, EDS was a significant predictor for poor sleep in chronic TMD patients. EDS was more prevalent in chronic TMD patients than in healthy controls (19.7% vs. 12.8%, *p* < 0.05), and its OR value for poor sleep quality was 1.069. The EDS prevalence in the present study was higher than the prevalence among the general population (12–16%) [52], and lower than 28.57% of TMD patients, the rate previously reported [27]. The discrepancy may have occurred due to differences in age distribution, race, and method of study. A significant proportion of the general population, as well as in patients with sleep problems or chronic pain, may suffer from EDS for a variety of causes, not pathological mechanisms. In older adults, they are prone to have daytime napping and EDS and the presence of comorbid conditions such as chronic pain, sleep disorders, and frequent nighttime urination breaks [53]. Diminished melatonin secretion and a reduced circadian modulation of rapid-eye-movement sleep and less pronounced day-night differences in the lower alpha activity occurs in the older group [54]. Furthermore, females are more likely than males to have more trouble sleeping at night and experience EDS [55]. Thus, EDS in chronic TMD may have different underlying mechanisms of a homeostatic drive for sleep and pain control systems according to age and sex. Hence, it is necessary to consider age-and sex-related differences in chronic TMD patients to obtain accurate results translation.

The high likelihood of OSA was not the significant predictor for poor sleep quality in chronic TMD patients. The STOP-Bang questionnaire is a great tool to easily identify patients with suspected OSA [29]. However, traditionally, polysomnography (PSG) in a sleep laboratory has been considered as a standard tool for the diagnosis of OSA [56]. Once OSA is identified, these patients need a definite diagnosis using the PSG. There is a limit to the extended interpretation of the results of the sleep quality and high risk of OSA in patients with chronic TMD, as the relationship between OSA and TMD. Of course, a bidirectional association between OSA and TMD have been suggested [28, 57]. Although the clear mechanism has not been identified, oral appliances used in the treatment of OSA may be the cause of TMD with continuous mandibular advancement while sleeping [58]. Sleep bruxism, a major risk factor for TMD, may be linked to OSA through the sleep-related arousal reactions [59]. The link between sleep bruxism and OSA has been studied at various ages [60–62], which could potentially suggest a link between TMD and OSA. Sleep bruxism and PSG were not covered in this study, but will be fully addressed in subsequent studies.

Headache attributed to TMD was associated with an increase of PSQI global scores, and a significant predictor for poor sleep. As headaches are a common symptom of accompanying TMD, few researches have been done on the nature of headache attributed to chronic TMD. TMD patients with headache reported significantly higher levels of pain and mandibular dysfunction than patients with only TMD [63]. Headache can promote sleep disturbances, and sleep disturbances can also precede or trigger a headache attack [64]. Moreover, sleep deterioration has been associated with an increased risk for headaches, and in individuals with chronic headaches, shorter sleep duration has been associated with more severe pain [65]. Clearly, the underlying pathophysiology contributing to the close association and complex relationship among headache attributed to TMD, headache disorders, and sleep disorders are not fully explained. There may be complex bidirectional relationships, and can be explained by peripheral and central sensitization, malfunctions of neuroendocrine, immune, and vascular system, and even gene polymorphism.

Myofascial pain was also associated with poor sleep quality in chronic TMD patients. Similar to the present study, the substantial influence of myofascial pain on poor sleep quality in patients with TMD was documented [66]. Furthermore, according to the TMD diagnostic subgroups, and the impact on quality of sleep and the symptom severity can vary. TMD patients with muscle-derived or myofascial pain exhibit more advanced stages of depression and somatization than patients diagnosed with TMJ disc displacement, a joint-derived problem [67]. Other researchers also observed a higher impact in patients with myogenous complaints than those with disc disorders [68, 69]. While joint pain is characterized by a well-defined inflammatory process, chronic muscle pain presents with enigmatic pathophysiologic mechanisms, of which central sensitization is the common factor unifying these conditions [70]. In addition, females have more pain and widespread pain in more body areas than males, which may be related to their worse quality of sleep [71]. Therefore, depending on the subgroup of TMD, the mechanisms by which TMD signs and symptoms occur are different, and further investigation is needed on the effects on sleep quality.

Finally, the quality of sleep was lower in chronic TMD patients with multiple diagnoses than in patients with a single diagnosis. According to Gil-Martínez et al., patients with mixed pain, having arthrogenous and myogenous origin simultaneously, showed greater craniomandibular and neck disability than patients diagnosed with chronic joint pain or muscle pain only [72]. Patients with headache and TMDs reported significantly higher levels of pain and disability compared to patients with only TMDs [63]. These findings can be interpreted as increasing the number of TMD subgroup diagnoses can increase the severity of TMD symptoms. Overall, chronic TMD symptoms and multiple diagnoses may have a bidirectional cross-correlation, which can impair sleep quality. Chronic TMD, especially myofascial pain, headaches attributed to TMD, and sleep disturbance factors, may share the mechanism of occurrence and exacerbation.

Limitations of this study include the case-control study design, which cannot address a causal direction of effects and suggests only associations/correlations between the variables. In addition, the DC/TMD axis II instrument was not accompanied due to limitations of our experimental setup. Compared to objective measurements, such as PSG or oximetry, PSQI addresses a longer time frame and is suitable for large scale research [73]. Moreover, a comparison of validity with respiratory indices from polysomnographic recordings has been made for PSQI with some degree of success [74]. However, a study design with repeated PSG may be more powerful in detecting phase-related differences. Instead of using PSG, we used self-assessment measures of sleep due to feasibility and convenience, especially because of large sample size. PSG is an objective measure of biophysiologic sleep parameters, so we are planning further studies to expand these findings with PSG. In addition, to get a deeper understanding of the relation between chronic TMD and poor sleep quality, we need to investigate their biopsychosocial aspects; however, this study did not evaluate psychological distress in patients with chronic TMD. Further studies on the psychological aspects of chronic TMD patients are ongoing.

## Conclusions

The strength of this study is a comprehensive analysis of how various issues affect poor sleep quality among many factors. There have been studies investigating sleep quality using PSQI in TMD cases [13, 75]. In the present study, chronic TMD patients suffered more from impaired sleep than healthy control subjects, and poor sleep was associated with multiple comorbid symptoms. Thus, assessing sleep quality should be a routine part of the diagnostic work-up of chronic TMD patients. Furthermore, a multidisciplinary management approach is needed to address all the factors in addition to sleep that modulate pain experience. In the diagnosis and treatment of chronic TMD, a fragmented field of view is not suitable, and a multidisciplinary approach involving experts in neurology, endocrinology, gerontology, and psychology, in addition to orofacial pain experts, is required. The results from this study will help to establish strategies for individual treatment and management of chronic TMD patients.

## Data Availability

Since these are patient data, if there is a request for data disclosure, KHU-IRB will discuss it before disclosure
